# POLICY SERIES: LET’S CONNECT: LINKING RESEARCH TO POLICY IN FAMILY CAREGIVING

**DOI:** 10.1093/geroni/igad104.0824

**Published:** 2023-12-21

**Authors:** Lauren Bangerter, Michael Wittke, Fawn Cothran

## Abstract

There is a need in the field of family caregiving to bridge the gap between academic research and policy translation by providing interdisciplinary caregiving researchers the skills and training they need to ensure their research is relevant to today’s policy discussions on family caregiving at the state and national levels. Quality research rests on sound institutional processes and methodological practices. This session will explore opportunities to elevate caregiving research for advocacy and policy audiences and how to inform policymakers about cutting-edge caregiving research across disciplines - particularly by advancing goal 5 within the National Family Caregiver Strategy - which calls for a stronger research and data collection infrastructure. We will offer caregiving researchers the best approaches for integrating policy research and implementation science approaches into their work as well as tactics to help sharpen their skills in messaging caregiving research for policy audiences, writing for a variety of audiences and platforms, and presenting data for policy and advocacy audiences. Presenters will cover topics such as facilitating additional research by public and private institutions by standardizing, expanding, and improving data collection by federal agencies; improving data collection on family caregiver health conditions and risk factors; improving data collection on family caregivers of people with multiple conditions; constructing additional channels on the local level that can gather detailed patient and family caregiver experience data to inform public health programming and policy development; and disaggregating data on diverse family caregiver populations to address the lack of segmentation and intersectionality in family caregiver research.

